# Effects of video-based training on anticipation and decision-making in football players: A systematic review

**DOI:** 10.3389/fnhum.2022.945067

**Published:** 2022-11-10

**Authors:** Jie Zhao, Qian Gu, Shuo Zhao, Jie Mao

**Affiliations:** ^1^College of Sports Engineering and Information Technology, Wuhan Sports University, Wuhan, China; ^2^School of Physical Education, Shandong University, Jinan, China; ^3^Shandong Football Management Center, Jinan, China

**Keywords:** video-based training, decision-making, anticipation, performance, football

## Abstract

The training of athletes’ anticipation and decision-making skills has received increasing attention from researchers, who developed and implemented training programs to achieve this. Video-based training (VBT) has become a popular method in anticipation and decision-making skills training. However, little is known about the benefits of implementing VBT in soccer. This systematic review considered the results of studies on VBT aiming to develop decision-making and anticipation skills in football players, and analyzed its effects. Literature published up to March 2022 was systematically searched on the scientific electronic databases Web of Science, PubMed, Scopus, SportDiscus, and Google Scholar. In total, 5,749 articles were identified. After screening the records according to the set exclusion and inclusion criteria, ten articles were considered eligible, including six longitudinal studies and four acute studies. Eight of the ten included studies (80%) showed that VBT group performance in anticipation or decision-making skills was significantly better at post-test than at pre-test, as evidenced by improvements in response accuracy (RA), response times (RT), mean distance scores (MDS) and passing decision-making performance. In six studies that included the no video-based training (NVBT) group, results showed that athletes in the VBT group performed better in anticipation or decision-making skills than in the NVBT group, as evidenced by improvements in RA and RT performance. The studies used different methods for VBT, both explicit and implicit training effectively improved participants’ anticipation and decision-making skills. In addition, the implementation of the “first-person” perspective (i.e., the player’s perspective) and virtual reality (VR) improved the presentation of video stimuli, effectively improving anticipation and decision-making. The findings of this review suggest that VBT is beneficial in developing anticipation and decision-making judgments in football players. However, some findings were inconsistent with previous studies due to differences in intervention duration and experimental protocols, and further studies are needed. Furthermore, future research should actively seek to design appropriate retention tests and transfer tests to truly understand the benefits of VBT for athletes.

## Introduction

Perceptual-cognitive skills are considered to be executive functions that regulate athletic performance (Vestberg et al., 2012), including visual search (Vaeyens et al., 2007a), anticipation (Muller and Abernethy, 2012), decision-making (Denardi et al., 2017), and pattern recognition (Williams et al., 2012). Perceptual-cognitive skills have been shown to be a defining characteristic of expert performance (Mann et al., 2007). In other words, excellent motor perceptual-cognitive skills promote the formation and development of motor skills, and high levels of spatio-temporal perceptual-cognitive skills can effectively improve performance (Savelsbergh and Van der Kamp, 2000). For example, in a sport like soccer, the opportunity for player action can easily be surrounded (Fajen et al., 2009). Players must move their heads, bodies, and eyes to perceive their surroundings and calibrate their positions, those of their opponents, and those of their teammates (Fradua et al., 1996; Kim et al., 2005). Make the most favorable decision for subsequent actions based on the current situation (Bennett et al., 2019). The perceptual-cognitive processes of anticipation and decision-making are key skills related to performance (Baker et al., 2003). Anticipation is the ability to recognize the outcome of other athletes’ movements before they are performed (Williams et al., 2002). Decision-making is the process of finding, differentiating, comparing, and finally choosing a course of action by an individual when cognitively processing a decision-making phenomenon in an uncertain and complex dynamic situation (Causer and Ford, 2014; Silva et al., 2020). Research has shown that experts demonstrate superior anticipation and decision-making skills compared to novices (Mann et al., 2007), allowing them to make decisions faster, better, and more intuitively (Vaeyens et al., 2007b; Roca et al., 2012). Significant differences in the performance of experts and novices in anticipation and decision-making skills help distinguish athletes with different skill levels (Del Villar et al., 2007; Vítor de Assis et al., 2021).

Anticipation and decision-making skills are important requirements for soccer players (Bennett et al., 2019). Because soccer comprises variability and uncertainty (Romeas et al., 2016). The game is intense, the situation on the field is fluid, and players have to react promptly and accurately according to the situation (Rouwen et al., 2005). For example, in soccer, penalty kicks are typically taken at speeds in excess of 75 km/h, which gives goalkeepers only 400 ms to intercept the ball (Kuhn, 1988). With such time constraints, goalkeepers must concentrate on the most important events or sources of information to effectively respond and execute more successful interceptions (Cañal-Bruland et al., 2005). However, in soccer training, coaches tend to focus on physical and tactical skills, while anticipation and decision-making skills, which are often seen as important issues, are rarely trained systematically (Murgia et al., 2014).

Training of athletes in anticipation and decision-making skills typically includes methods such as video-based training (VBT) (Nelson et al., 2014) and game-based training (Davids et al., 2013). Game-based training involves athletes simulating the decision-making process of the game through self-guided discovery (Gabbett et al., 2009; Light et al., 2014). This approach focuses comprehensively on the interaction between tactical knowledge and skill execution, and is an effective means of improving anticipation and decision-making skills (O’Connor et al., 2017). However, this approach is limited by the number of games and the difficulty of organizing and managing efficient games (Kittel et al., 2020). VBT is a common method to overcome these limitations and effectively improve anticipation and decision-making (Engelbrecht et al., 2016). VBT was defined as a specific practice phase in which video is used to present stimuli that require participants’ perceptual-cognitive responses (Larkin et al., 2015; Hadlow et al., 2018). Approaches include viewing and simulating video sequences of matches (Ward and Williams, 2003; Farahani et al., 2020), temporal occlusion (Smeeton et al., 2005; Brenton et al., 2016), occlusion of action sequences, feedback to participants on the accuracy of test results (Gorman and Farrow, 2009; Nelson et al., 2014), and directing attention direction through video information (Hagemann et al., 2006). VBT allows learners to practice without actually performing the skill (Larkin et al., 2014). Especially in sports such as soccer, which require prolonged participation, this approach can accelerate the learning of expertise and speed up the process of perception-cognitive development (Page et al., 2019). By using video training, coaches can control some scenarios according to specific needs, allowing injured players to participate in the training and avoiding increasing the physical load of the athletes (Starkes and Lindley, 1994; Horn et al., 2002). Therefore, this form of practice is the most common way to develop athletes’ anticipation and decision-making skills.

The researchers noted that VBT tasks need to maintain as much ecological validity as possible (Silva et al., 2021). To achieve this, the proximity of the video simulation environment to real-life should be taken into account when designing training programs (Raab et al., 2019). Hays (1989) used the term “fidelity” to the comparability between simulated tasks and the real world. Common VBT use broadcast video of matches (Craig, 2013). This approach lacks fidelity and is often criticized (Broadbent et al., 2015). To increase the representativeness of existing training tasks, virtual reality (VR) is beginning to be incorporated into training (Panchuk et al., 2018; Page et al., 2019). VR provides a greater sense of immersion for viewers by increasing the visual correspondence of video simulations (Kittel et al., 2020). VR has been identified as a new VBT method (Vignais et al., 2015). In addition, most of the early studies attempted to explicitly teach participants to focus on “information-rich” areas, providing guiding information for their perceptual-cognitive training (Abernethy et al., 1999; Savelsbergh et al., 2002). This type of training is referred to as explicit training. Hanvey (1999) informed goalkeepers of the rules and cues associated with kick position, and their ability to anticipate the direction of the shot is improved through explicit training. However, subsequent studies have tended to use implicit training (Farrow and Abernethy, 2002; Jackson and Farrow, 2005; Masters et al., 2008). Implicit training promotes guiding participants to seek out key sources of information without explicitly stating the relationship between visual stimuli and changes in response requirements (Shafizadeh and Platt, 2012). Magill (1998) and Farrow and Abernethy (2002) have argued that implicit training can more effectively in improving perceptual-cognitive skills and produce lasting perceptual-cognitive learning effects. However, there is still some debate as to which training method is better at improving perceptual-cognitive skills.

Furthermore, the study of perceptual-motor performance from an ecological perspective emphasizes that motor behavior is a coupling between perceptual and motor systems (Davids et al., 2006). In contrast, most VBT does not include complex motor responses (Hagemann et al., 2006; Gorman and Farrow, 2009; Brenton et al., 2016). This limits the link between perceptual and motor processes. In studies of perceptual-cognitive skills, Dicks et al. (2010) examined the motor performance of soccer goalkeepers in laboratory conditions and field conditions and demonstrated that information extraction was different in perception-action coupled and uncoupled tasks. In the perception-action uncoupled condition, the goalkeeper focused more on attending to the action information of the penalty taker rather than the position of the ball. In contrast, in the perception-action coupled task, the goalkeeper pays attention to both the relative actions of the penalty taker and the position of the ball. There are also some studies that pure perceptual-cognitive training is equally effective compared to perceptual-motor training (Farrow and Abernethy, 2002; Hagemann and Memmert, 2006; Ranganathan and Carlton, 2007). Controversy remains regarding the effectiveness of perceptual-cognitive training in the context of separation of action and perception. Subsequent studies have made improvements by adding transfer tests to the task (Gabbett et al., 2008; Rosalie and Müller, 2012; Lorains et al., 2013; Brenton et al., 2019). Transfer tests were used to measure whether performance improvements transfer to real-world competition situations (Starkes and Lindley, 1994). These studies reported that perceptual-cognitive training that did not involve motor responses could improve real-world motor performance (Gabbett et al., 2008; Rosalie and Müller, 2012; Lorains et al., 2013; Brenton et al., 2019). For this reason, it is important that the measurement effect is transferable to the competition (Smeeton et al., 2005; Williams and Ward, 2007). In addition, the researchers suggest that retention tests should be included to determine whether there is a potential lasting benefit to VBT (Schmidt et al., 2018).

In recent years, as research into athletes’ perceptual-cognitive skills has continued, there have been some studies showing that VBT can enhance athletes’ anticipation and decision-making skills. However, little is known about the benefits of implementing VBT in soccer. Furthermore, to our knowledge, only Larkin et al. (2015) have reviewed VBT to enhance athletes’ perceptual-cognitive skills, summarizing the effectiveness of the video-based approach to enhance decision-making skills before 2013. There is a lack of systematic summary of post-2013 research, especially on the implementation of VBT in soccer. Therefore there is a need for a systematic review of this research topic. The purpose of this systematic review is to summarize the effectiveness of VBT to develop anticipation and decision-making judgments in football players, and to analyze various approaches to VBT.

## Materials and methods

Our systematic review was conducted following the Preferred Reporting Items for Systematic Reviews and Meta-Analyses (PRISMA) (Page et al., 2021). We used the Population, Intervention, Comparison, Outcome (PICO) tool to help formulate the research questions (da Costa Santos et al., 2007). “Does VBT improve soccer players’ anticipation and decision-making skills more than NVBT or other training? “

### Search strategy

We searched the following electronic databases for studies published up to March 2022: Web of Science, PubMed, Scopus, SportDiscus, and Google Scholar. To search the relevant literature as comprehensively as possible, we developed the following on the basis of the above definitions. Search strategy: #1 (“video-feedback” OR “video-based” OR “video-based training” OR “video training”); #2 (“visual function” OR “executive function” OR “attention”); #3 (“decision-making” OR “decision-making training” OR “anticipation” OR “perceptual training” OR “cognitive training” OR “perceptual-cognitive training” OR “Perceptual functions”); #4 (“football” OR “soccer” OR “penalty kick” OR “goalkeepers”); (#1 OR #2 OR #3) AND #4.

### Inclusion criteria

The inclusion and exclusion criteria were determined by two authors (JZ and QG) and independently reviewed and evaluated. The inclusion criteria were as follows: (i) the participant group was football players; (ii) the content of the paper focused on training to improve players’ perceptual-cognitive skills (i.e., decision-making or anticipation); (iii) video was used as the training stimulus or task; (iv) the article provided information about samples and experimental methods/procedures (e.g., describing data collection procedures, experimental methods, instrumentation, and measures); (v) the article reported the relevant findings of the training; (vi) controlled experiments (interventions with a control group); (vii) intervention studies. The exclusion criteria were as follows: (i) lack of experimental methods and research outcomes; (ii) articles not peer-reviewed; (iii) articles in languages other than English; (iv) non-intervention studies; and (v) designs without control groups.

### Extraction of data

Two authors (JZ and QG) independently extracted the following information from the included studies: (i) publication year, location; (ii) number, age, gender, and exercise experience of participants; (iii) measures of intervention, duration; and (iv) study results. Performance outcomes include response accuracy (RA), response times (RT), mean distance score (MDS), and passing decision-making performance. RA is used to test the effect of the intervention on the athlete’s anticipation or decision-making RA. RT is used to test the effect of the intervention on the athlete’s anticipation or decision-making response times. MDS indicates the distance between the athlete’s judged kick position in the test and the actual kick position. Passing decision-making performance is used to test the impact of an intervention on the accuracy of an athlete’s technical action in passing. We retrieved RA as a measure of anticipation or decision-making performance if the findings indicated that VBT had a differential impact on anticipation or decision-making performance (e.g., RA improved but RT decreased). The effects of different VBT modalities on perceptual-cognitive skills, including explicit training and implicit training, were also investigated.

### Methodological quality

The methodological quality of the included studies was evaluated independently by two authors (JZ and QG). In cases of disagreement, a third author (JM) deliberated until consensus was reached. The methodological quality of the included studies was assessed using the Physiotherapy Evidence Database (PEDro) scale (Elkins et al., 2013). The scale consists of 11 items, namely, eligibility criteria, randomization, concealed allocation, baseline equivalence, blinding of subjects, blinding of instructors, blinding of assessors, retention above 85%, intention to treat analysis, between-group comparison, point measures and measures of variables. Items with a clear description were given 1 point and those without a clear description were given 0 points.

## Results

### Study selection

**FIGURE 1:**
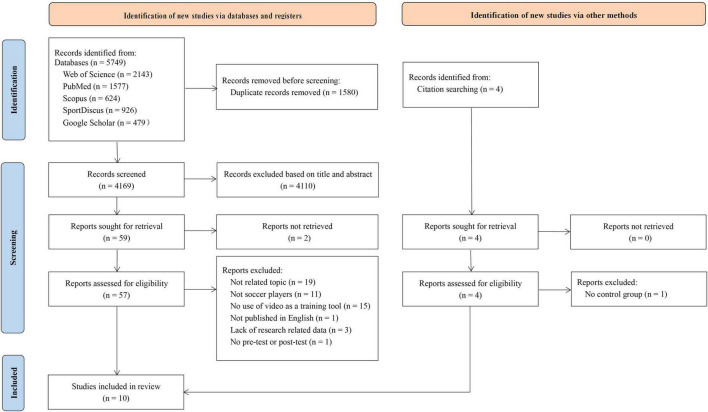
Flowchart of search and study selection according to PRISMA guidelines.

Our search of the five scientific electronic databases identified 5,749 titles, from which duplicate and irrelevant articles were removed, leaving 4,173 papers which were filtered by title and abstract. Of these, 61 articles met the inclusion criteria. Two authors (JZ and QG) excluded 51 articles after independently assessing the 61 articles using the predetermined inclusion and exclusion criteria. The remaining 10 studies were eligible for inclusion in this study. Figure 1 shows the flowchart for the search and selection.

### Characteristics of included studies

#### Overview

Overall, 10 studies were included. Studies were divided into two categories based on the type of intervention: (i) acute studies, defined as interventions lasting less than 24 h (*n* = 4) (Poulter et al., 2005; Nunez et al., 2009; Javier Nunez et al., 2010; Shafizadeh and Platt, 2012); and (ii) longitudinal studies, defined as interventions duration ≥ 24 h (*n* = 6) (Gabbett et al., 2008; Savelsbergh et al., 2010; Ryu et al., 2013; Murgia et al., 2014; Nimmerichter et al., 2016; Fortes et al., 2021). The characteristics of the included studies are summarized in Tables 1, 2, respectively. The included studies provided a total of 300 participants (198 males: 66% and 102 females: 34%). The sample size for each study ranged from 16 to 48. The participants ranged in age from 14 to 25 years. These studies were conducted using both pre-test and post-test research designs.

**TABLE 1:** Characteristics of the included longitudinal studies.

Study (authors, publication year, methodological quality, location)	*N*	Skill level	VBT		NVBT	Intervention duration/ session length	Testing times	Results
							
			(1) Gender (2) Age (years) (3) Playing experience (years) (4) Interventions	(1) Gender (2) Age (years) (3) Playing experience (years) (4) Interventions	(1) Gender (2) Age (years) (3) Playing experience (years)			
Gabbett et al. (2008) 6/12 Australia	16	Elite	(1) 8F/8 (2) 18.3 ± 2.8 (3) NA	None	(1) 8F/8 (2) 18.3 ± 2.8 (3) NA	4 weeks	Pre-test; post-test; transfer test	RA of tasks was significantly improved in the VBT group at post-test (*p* = 0.05), and RA of tasks in the NVBT group was not significantly different (*p* > 0.05). Passing, dribbling, and shooting decision-making skills improved in the VBT group in the transfer test, while there was no change in the NVBT group.
Savelsbergh et al. (2010) 7/12 Netherlands	30	Novice	(1) NA (2) 22.0 ± 3.6 (3) 5.7 ± 3.5 (recreational) (4) IT	(1) NA (2) 22.0 ± 3.6 (3) 5.7 ± 3.5 (recreational) (4) UT	(1) NA (2) 22.0 ± 3.6 (3) 5.7 ± 3.5 (recreational)	6 days	Pre-test; post-test	At pre-test, there was no significant difference in RA between the groups of tasks, and the IT group (RA increased by 12.6) performed better at post-test compared to the UT (RA decreased by 2.7) and NVBT groups (RA increased by 3.7). RT of the tasks in the IT group increased at post-test compared to pre-test, with no significant change in the other groups.
Ryu et al. (2013) 7/12 China (Hong Kong)	28	Novice	(1) 9M/9 (2) 22.6 ± 2.7 (3) 0 (4) IT	(1) 10M/10 (2) 22.6 ± 2.7 (3) 0 (4) UT	(1) 9M/9 (2) 22.6 ± 2.7 (3) 0	1 week	Pre-test; post-test; retention test	RA improved significantly better in the IT group than in the NVBT group without impairing RT (p < 0.001). RA improved in the IT and UT groups, while the improvement was better in the IT group (p < 0.001). In the retention test, RA was better in the IT group than in the other groups.
Murgia et al. (2014) 7/12 Italy	38	Elite	(1) 13M/13 (2) 16.0 ± 1.9 (3) 9.3 ± 2.6	(1) 13M/13 (2) 16.0 ± 1.9 (3) 9.3 ± 2.6 (4) VST	(1) 12M/12 (2) 16.0 ± 1.9 (3) 9.3 ± 2.6	8 weeks	Pre-test; post-test	In the simulated penalty kick task, RA in the horizontal and vertical directions was significantly improved in the VBT group (p < 0.001), but not in the VST and NVBT groups.
Nimmerichter et al. (2016) 6/12 Austria	34	Elite	(1) 18M/18 (2) 14.4 ± 0.1 (3) 3–5	None	(1)16M/16 (2) 14.4 ± 0.1 (3) 3–5	6 weeks	Pre-test; post-test	RA and RT of tasks were significantly improved in the VBT group at post-test (p < 0.001, p = 0.006), and RA was significantly improved by 34% and RT by 24%. While there was no significant change in the NVBT group (p = 0.125, p = 0.297).
Fortes et al. (2021) 8/12 Brazil	26	Elite	(1) 13F/13 (2) 15.4 ± 0.3 (3) 5.0 ± 1.2 (4) VST	(1) 13F/13 (2) 15.4 ± 0.3 (3) 5.0 ± 1.2 (4) VRT	None	8 weeks	Pre-test; post-test	Passing decision-making skills were improved in the on-field game assessment (p < 0.005), and the VRT group showed greater improvement compared to the VST group (p < 0.005).

F, female; IT, implicit training; M, male; NA, not available; NVBT, no video-based training; RA, response accuracy; RT, response times; UT, unguided training; VBT, video-based training; VRT, virtual reality training; VST, video-screen training.

**TABLE 2:** Characteristics of the included acute studies.

Study (authors, publication year, methodological quality, location)	N	Skill level	VBT				NVBT	Testing times	Results
						
			(1) Gender (2) Age (years) (3) Playing experience (years) (4) Interventions	(1) Gender (2) Age (years) (3) Playing experience (years) (4) Interventions	(1) Gender (2) Age (years) (3) Playing experience (years) (4) Interventions	(1) Gender (2) Age (years) (3) Playing experience (years) (4) Interventions	(1) Gender (2) Age (years) (3) Playing experience (years)		
Poulter et al. (2005) 7/12 United States	48	Novice	(1) 12F/12 (2) 20.5 ± 4.7 (3) 0 (4) ET	(1) 12F/12 (2) 20.5 ± 4.7 (3) 0 (4) IT	(1) 12F/12 (2) 20.5 ± 4.7 (3) 0 (4) VST	(1) 12F/12 (2) 20.5 ± 4.7 (3) 0 (4) UT	None	Pre-test; post-test	The ET and VST groups showed significant improvement in RA in the horizontal direction at post-test (*p* < 0.01), with greater improvement in the ET (RA increased by 14.86) than in the VST (RA increased by 13.19), and no significant change in the IT and UT groups (*p* = 0.20). There was no significant improvement in RA in the vertical direction for the four groups (*p* > 0.05).
Nunez et al. (2009) 5/12 Spain	20	Elite; novice	(1) NA (2) 25.7 ± 4.2 (3)≥10 (4) ET	(1) NA (2) 22.1 ± 2.5 (3) 0 (4) ET	(1) NA (2) 25.7 ± 4.2 (3)≥10 (4) UT	(1) NA (2) 22.1 ± 2.5 (3) 0 (4) UT	None	Pre-test; post-test	At post-test, the ET elite group had a faster RT compared to the other groups, RT reduced by 102 s. RA was higher in the two ET groups than in the two UT groups, and there was no significant difference between the ET novice group and the ET elite group.
Javier Nunez et al. (2010) 6/12 Spain	32	Elite	(1) 8M/8 (2) 23.2 ± 1.8 (3)≥10 (4) ET	(1) 8M/8 (2) 23.2 ± 2.5 (3) ≥10 (4) IT	(1) 8M/8 (2) 23.2 ± 2.3 (3)≥10 (4) UT	None	(1) 8M/8 (2) 23 ± 2.2 (3)≥10	Pre-test; post-test; retention test	In the simulated penalty kick task, RA was significantly improved in the ET group, with RA increasing by 23.1%, and no significant change was observed in the other groups. RT was significantly increased in the ET and UT groups, and increased by 55 and 112 s, respectively. In both retention tests, RA and RT were higher in the ET group than in the other groups.
Shafizadeh and Platt (2012) 7/12 United States	28	Novice	(1) 14M/14 (2) 19 ± 2.2 (3) ≤2.5 (4) ET	(1) 14M/14 (2) 19 ± 2.2 (3) ≤2.5 (4) UT	None	None	None	Pre-test; post-test	In the simulated penalty kick task, MDS was improved (*p* < 0.03), and the ET group showed greater improvement compared to the UT group.

ET, explicit training; F, female; IT, implicit training; M, male; MDS, mean distance scores; NA, not available; NVBT, no video-based training; RA, response accuracy; RT, response times; UT, unguided training; VBT, video-based training; VST, Video-screen training.

#### Longitudinal studies

In the six longitudinal studies (Gabbett et al., 2008; Savelsbergh et al., 2010; Ryu et al., 2013; Murgia et al., 2014; Nimmerichter et al., 2016; Fortes et al., 2021), the length of the interventions ranged from 6 days to 8 weeks. Four studies involved elite athletes (Gabbett et al., 2008; Murgia et al., 2014; Nimmerichter et al., 2016; Fortes et al., 2021) and two studies involved novices (Savelsbergh et al., 2010; Ryu et al., 2013). One study involving elites used innovative interactive home training that allowed participants the freedom to schedule training cycles without any experimenter supervision (Murgia et al., 2014). The remaining studies were conducted in a laboratory setting and required experimenter supervision or the use of specific equipment (Gabbett et al., 2008; Savelsbergh et al., 2010; Ryu et al., 2013; Nimmerichter et al., 2016; Fortes et al., 2021). Two of the six studies asked participants to perform video training from a “third-person” perspective (i.e., the broadcast perspective) (Gabbett et al., 2008; Murgia et al., 2014), and four asked participants to watch football videos from a “first-person” perspective (i.e., the player’s perspective) (Savelsbergh et al., 2010; Ryu et al., 2013; Nimmerichter et al., 2016; Fortes et al., 2021). One study conducted retention tests to examine the effects of skill retention after the training period (Ryu et al., 2013). One study conducted transfer tests to measure the translation of performance improvements into a realistic competition environment (Gabbett et al., 2008).

#### Acute studies

Among the four acute studies (Poulter et al., 2005; Nunez et al., 2009; Javier Nunez et al., 2010; Shafizadeh and Platt, 2012), one study involved elites athletes (Javier Nunez et al., 2010), two studies involved novices (Poulter et al., 2005; Shafizadeh and Platt, 2012), and one study involved both novices and elites (Nunez et al., 2009). The four studies were conducted in the laboratory and the training program was supervised (Poulter et al., 2005; Nunez et al., 2009; Javier Nunez et al., 2010; Shafizadeh and Platt, 2012). In addition, all four studies required participants to watch football videos from a “first-person” perspective (Poulter et al., 2005; Nunez et al., 2009; Javier Nunez et al., 2010; Shafizadeh and Platt, 2012). One study also conducted retention tests (Javier Nunez et al., 2010).

### Methodological quality of included studies

The rating scores for the methodological quality of each study based on the PEDro scale are presented in Tables 1, 2, with a mean score of 6.6 and a range of 5–8 (see Supplementary Table for details).

## Study findings

### Longitudinal studies

Five of the six longitudinal studies (83.3%) showed significant improvements in anticipation or decision-making skills performance in the VBT group at post-test than at pre-test (Gabbett et al., 2008; Ryu et al., 2013; Murgia et al., 2014; Nimmerichter et al., 2016; Fortes et al., 2021). Five studies that included the NVBT group showed that the VBT group outperformed the NVBT group in anticipation or decision-making skills at post-test (Gabbett et al., 2008; Savelsbergh et al., 2010; Ryu et al., 2013; Murgia et al., 2014; Nimmerichter et al., 2016). In addition, the results of one retention test (Ryu et al., 2013) and one transfer test (Gabbett et al., 2008) were reported. The retention test result showed that the VBT group maintained short-term skill improvements over the NVBT group (Ryu et al., 2013), and the transfer test result reported better transfer of skills in the VBT group than in the NVBT group (Gabbett et al., 2008).

Seven performance outcomes were reported in six longitudinal studies. Four studies included RA as a performance outcome (Gabbett et al., 2008; Savelsbergh et al., 2010; Ryu et al., 2013; Murgia et al., 2014), one study included RA and RT as performance outcomes (Nimmerichter et al., 2016), and one study included passing decision-making skills as a performance outcome (Fortes et al., 2021). Six of the seven outcomes reported significantly improved performance (RA, RT, and passing decision-making skills) in the VBT group and better performance than the NVBT group at post-test (Gabbett et al., 2008; Ryu et al., 2013; Murgia et al., 2014; Nimmerichter et al., 2016; Fortes et al., 2021). One outcome reported better performance (RA) in the VBT group than in the NVBT group at post-test (Savelsbergh et al., 2010). In addition, one transfer test included passing, dribbling, and shooting decision-making skills as performance outcomes (Gabbett et al., 2008). The results showed that the athletes in the VBT group performed better in passing, dribbling and shooting decision-making skills than the athletes in the NVBT group.

Two of the six longitudinal studies examined the effects of implicit training on penalty kick anticipation skills in novice participants (Savelsbergh et al., 2010; Ryu et al., 2013). These two studies reported better anticipation performance in the implicit training group than in the group that did not receive guidance (Savelsbergh et al., 2010; Ryu et al., 2013). In addition, one study included retention tests and showed that the implicit training group maintained short-term skill improvements over the group that did not receive guidance (Ryu et al., 2013). One other study used an immersive 3D video stimulus with elite participants (Fortes et al., 2021). The results showed that the immersive 3D video group had significantly improved passing decision-making skills compared to the 2D video group.

### Acute studies

Three of the four acute studies (75%) showed significant improvements in anticipation or decision-making skills performance in the VBT group at post-test than at pre-test (Poulter et al., 2005; Javier Nunez et al., 2010; Shafizadeh and Platt, 2012). One study that included the NVBT group showed that the VBT group outperformed the NVBT group in anticipation or decision-making skills at post-test (Javier Nunez et al., 2010). In addition, the results of one retention test were reported, showing that the retention of skills was superior in the VBT group than in the NVBT group (Javier Nunez et al., 2010).

Six performance outcomes were reported in four acute studies. One study included RA as a performance outcome (Poulter et al., 2005), two studies included RA and RT as performance outcomes (Nunez et al., 2009; Javier Nunez et al., 2010), and one study included MDS as a performance outcome (Shafizadeh and Platt, 2012). One of the six outcomes RA in the VBT group and outperformed the NVBT group at post-test (Savelsbergh et al., 2010). Four outcomes (RA, RT and MDS) in the VBT group (Poulter et al., 2005; Nunez et al., 2009; Shafizadeh and Platt, 2012). One outcome reported a decrease in performance (RT) in the VBT group (Savelsbergh et al., 2010).

Two of the four acute studies examined the effects of explicit training on the performance of penalty kick anticipation skills (Nunez et al., 2009; Shafizadeh and Platt, 2012). One study with novice participants showed an improvement in anticipation performance in the explicit training and the no guidance groups, with the explicit training group outperforming the no guidance group (Shafizadeh and Platt, 2012). One study with novice and elite participants found that the explicit training group performed better in anticipation skills compared to the group that did not receive guidance (Nunez et al., 2009). There was no significant difference in anticipation performance between novices and elites in explicit training.

Of the four acute studies, two compared the effects of implicit and explicit training on the performance of penalty kick anticipation skills (Poulter et al., 2005; Javier Nunez et al., 2010). The results of one study in which participants were novices, showed that the explicit training group improved performance on participant anticipation significantly, while the implicit training group showed no significant change (Poulter et al., 2005). One study with elite participants reported that explicit training improved participants’ anticipation performance better than implicit training (Javier Nunez et al., 2010). Retention tests were also conducted in this study and showed that the explicit training group was more effective than the implicit training group in terms of skill retention (Javier Nunez et al., 2010).

## Discussion

We review research on video-based perceptual-cognitive training to develop anticipation and decision-making skills in football players. The effectiveness of VBT to improve anticipation and decision-making judgments were analyzed and various training methods used for video tasks were also considered. The findings highlighted several key findings: (i) the available evidence tends to support the positive effects of VBT on improving the anticipation and decision-making skills in football players; (ii) VBT used training methods such as implicit and explicit training; (iii) researchers improved the presentation of video stimuli during training to effectively improve anticipation and decision-making.

### Video-based training to develop the effectiveness of anticipation and decision-making judgments

We reviewed ten studies, including six longitudinal studies (Gabbett et al., 2008; Savelsbergh et al., 2010; Ryu et al., 2013; Murgia et al., 2014; Nimmerichter et al., 2016; Fortes et al., 2021) and four acute studies (Poulter et al., 2005; Nunez et al., 2009; Javier Nunez et al., 2010; Shafizadeh and Platt, 2012). Of these, five studies assessed the effects of VBT on elite skill performance (Gabbett et al., 2008; Javier Nunez et al., 2010; Murgia et al., 2014; Nimmerichter et al., 2016; Fortes et al., 2021), four studies investigated the effects on novice skill performance (Poulter et al., 2005; Savelsbergh et al., 2010; Shafizadeh and Platt, 2012; Ryu et al., 2013), and one study examined both elite and novice athletes (Nunez et al., 2009). Eight of the ten included studies (80%) showed that performance in anticipation or decision-making skills was significantly better in the VBT group at post-test than at pre-test (Poulter et al., 2005; Gabbett et al., 2008; Javier Nunez et al., 2010; Shafizadeh and Platt, 2012; Ryu et al., 2013; Murgia et al., 2014; Nimmerichter et al., 2016; Fortes et al., 2021). Six studies that included the NVBT group reported better performance in anticipation or decision-making skills in the VBT group than in the NVBT group (Gabbett et al., 2008; Javier Nunez et al., 2010; Savelsbergh et al., 2010; Ryu et al., 2013; Murgia et al., 2014; Nimmerichter et al., 2016). Of these, four showed significant improvements in anticipation or decision-making skills performance in the VBT group, while only small but non-significant changes were found in the NVBT group (Gabbett et al., 2008; Javier Nunez et al., 2010; Murgia et al., 2014; Nimmerichter et al., 2016). Two showed that the VBT group performed better than the NVBT group on anticipation or decision-making skills at post-test (Savelsbergh et al., 2010; Ryu et al., 2013). In addition, a study investigating both elites and novices found no significant differences in skill performance between novices and elites (Nunez et al., 2009). VBT helped novices and elites in their anticipation and decision-making skills.

When assessing the effects of VBT on anticipation and decision-making skills in football players, researchers typically report RA and RT as performance outcomes. Eight studies reported RA outcomes and the outcomes showed an improvement in participants’ RA performance through VBT (Poulter et al., 2005; Gabbett et al., 2008; Nunez et al., 2009; Javier Nunez et al., 2010; Savelsbergh et al., 2010; Ryu et al., 2013; Murgia et al., 2014; Nimmerichter et al., 2016). In addition to accurate responses, football requires rapid responses. Three studies reported outcomes for RT (Nunez et al., 2009; Javier Nunez et al., 2010; Nimmerichter et al., 2016). Two of the three studies claimed that participants’ RT improved through VBT (Nunez et al., 2009; Nimmerichter et al., 2016). The remaining study found a decrease in participants’ RT performance (Javier Nunez et al., 2010). We found from this review that the researchers improved the accuracy of the participants’ decision-making by guiding them to valid information at key points, resulting in an increase in the time required to respond (Javier Nunez et al., 2010). However, it should be emphasized that VBT is not just about getting players to pay attention to the most useful information. It is also about getting players to learn how to time their attention so that they can accurately grasp the most useful sources of information as they become available (Abernethy et al., 1999; Savelsbergh et al., 2010).

Findings from the included studies showed that VBT can be beneficial in enhancing the anticipation and decision-making skills of football players. Football presents a complex, fluid, and unpredictable situation, and to initiate action quickly, players must concentrate on the most pertinent sources of information or significant occurrences (Harenberg et al., 2021). As a result, there is a high demand on the perceptual-cognitive skills of the athletes. VBT was found to help to promote perceptual-cognitive skills (Larkin et al., 2015), which may explain its advantages in training. In VBT, researchers typically show participants video sequences in which the video stops at a certain decision point, and participants are asked to make a judgment about the direction of the action (hitting, shooting, or throwing) (Williams and Grant, 1999). In this way, only specific elements are perceived, and their attention is drawn to the key stimuli, thus accelerating the development of perceptual mechanisms that help to accurately anticipate the opponent’s intention to act and make effective decisions (Nimmerichter et al., 2016).

VBT primarily assesses changes in performance before and after the intervention. However, improvement in performance may be a transient result, and retention tests are required at the end of the study to determine if there is a potentially lasting benefit of VBT. Only two of the included studies included retention tests after training (Javier Nunez et al., 2010; Ryu et al., 2013). These studies showed that VBT maintained short-term skill improvements. Of these, Ryu et al. (2013) conducted a retention test after 24 h and showed that the improvement in performance was maintained after the 24 h interval. Javier Nunez et al. (2010) conducted retention tests at intervals of 1 and 7 days. The results showed that improvements in performance were maintained at both 1 and 7 days, but the decline in acquisition performance gradually became larger as time increased.

Another important aspect of VBT is how performance improvements are transferred to real game situations. The key to this is to design a suitable transfer test for the training task (Lorains et al., 2013; Larkin et al., 2015). This is an important consideration in the study of perceptual-cognitive skills because athletes are ultimately measured by their performance on the court (Luis del Campo et al., 2015). Only one of the included studies included the transfer test to evaluate the likelihood of transferring training effects to real matches (Gabbett et al., 2008). For this purpose, the study organized a small-scale standardized training competition. The researchers coded the athletes’ decision-making skills on the field, and a sports scientist assessed the athletes’ passing, dribbling and shooting skills. The results of the assessment claimed that the VBT group performed significantly better than the NVBT group in passing, dribbling and shooting decision-making skills (Gabbett et al., 2008). The anticipation or decision-making skills learned through VBT can be applied to the pitch. However, in the other nine studies, the effect of improvement in participants’ anticipation and decision-making skills in real matches could not be determined. Although it has been shown that retention tests and transfer tests should be included in video-based tasks, this has rarely been considered. To enhance the evaluation and generalization of the results, it is reasonable to encourage future research to investigate this further.

### Different video-based training methods to develop the effectiveness of anticipation and decision-making judgments

Of the included studies, two examined the effects of explicit training on anticipation or decision-making skills (Nunez et al., 2009; Shafizadeh and Platt, 2012). The training involved the experimenter explicitly informing participants about the key cues of the stimulus, allowing participants to concentrate on relevant information and ignore irrelevant information (Nunez et al., 2009; Shafizadeh and Platt, 2012). Two examined the impacts of implicit training on the performance of anticipation or decision-making skills, where participants were asked to watch video clips with highlights and the researchers gave them no instructions other than encouraging them to follow the highlights (Savelsbergh et al., 2010; Ryu et al., 2013). The results reported that the implicit training group outperformed the group that did not receive guidance (Savelsbergh et al., 2010; Ryu et al., 2013). We conclude from these studies that explicit and implicit training is effective. Explicit and implicit training improves the athletes’ ability to recognize movement patterns, and allows them to direct their attention to task-relevant information, correctly capture key information in movement scenes, and make effective decisions based on the goals of the game (Gorman and Farrow, 2009; Ryu et al., 2013).

In addition, two of the included studies compared the impacts of explicit and implicit training on skill performance (Poulter et al., 2005; Javier Nunez et al., 2010). The results indicated that explicit training improved participants’ performance on anticipation or decision-making skills more than implicit training. These findings are inconsistent with previous studies by Magill (1998) and Smeeton et al. (2005) possibly due to the fact that these two studies were acute studies that should have taken into account the transient nature of the training phase. It is also worth noting that both studies had participants respond using a verbal response format, which may have also limited the improvement in the performance of the implicit training group (Poulter et al., 2005; Javier Nunez et al., 2010). Because this form of response does not include a motor response, this limits the connection between perception and motor processes (Davids et al., 2006).

In summary, the current results suggest that both explicit and implicit training improve participants’ anticipation and decision-making performance more than training without guidance. However, more research is required to identify which type of training produces better results on skill performance.

### Researchers improve the presentation of video stimuli to effectively enhance anticipation and decision-making

Two of the included studies viewed football video clips from a “third-person” perspective, using videos taken from a fixed position that did not simulate the perspective of the player in the game (Gabbett et al., 2008; Murgia et al., 2014). To improve the fidelity of the video simulation task, in eight studies the opponent was described from the perspective of the participants (Poulter et al., 2005; Nunez et al., 2009; Javier Nunez et al., 2010; Savelsbergh et al., 2010; Shafizadeh and Platt, 2012; Ryu et al., 2013; Nimmerichter et al., 2016; Fortes et al., 2021). These videos were one-on-one situations filmed by athletes wearing helmet cameras from a “first-person” perspective, which represents a dynamic “self-perception” of the game scene (Nimmerichter et al., 2016). “Self-perception” is interpreted as imitating the action of the game as realistically as possible and is key to effective performance (Roca et al., 2011).

Although researchers have worked hard to increase the fidelity of video simulations, discrepancies with real-life remain inevitable. VR can minimize this shortcoming and increase the representativeness of the task (Fortes et al., 2021), and is therefore beginning to be incorporated into training. VR is a 3D computer technology-based simulation that creates a virtual world with multiple sensory experiences, allowing athletes to immerse themselves in it and achieve direct interaction with the virtual environment (Banos et al., 2016). It is important to highlight that there are different types of VR, such as virtual video (Hohmann et al., 2016; Gray, 2017) and 360 VR (Panchuk et al., 2018; Page et al., 2019; Kittel et al., 2021). Virtual video enhances the visual stimulation of participants compared to 2D video, but the limitation of this approach is that participants cannot interact with the environment, which limits perception-action coupling (Vignais et al., 2015). Fortes et al. (2021) used 360VR to stimulate participants’ decision-making performance, and the training video was presented through a head-mounted display. 360VR allows participants to interact freely with the environment, adding a visual counterpart to the video simulation (Kittel et al., 2020). The results showed that passing decision-making skills were significantly improved in the 360VR group compared to the 2D video group. We recommend incorporating 360VR into training programs as the presentation in VR is closer to the perception on a real football pitch than 2D video and virtual video, are more immersive, maximize task representation, and effectively improve perceptual-cognitive skills.

### Practical implications

Perceptual-cognitive training is an increasingly important topic in the field of sports. The findings of this review found that VBT has a positive effect on improving anticipation and decision-making skills in football players. This approach accelerates the learning of expertise and the development of perceptual mechanisms, and is an effective means of enhancing anticipation and decision-making skills (Nimmerichter et al., 2016). In addition, athletes can train in situations where they are unable to physically train (e.g., injuries) with minimal requirements for equipment and facilities, and may even train at home (Murgia et al., 2014). With the development of video technology, the opportunity to design more immersive and interactive perceptual-cognitive training environments is increasing (Fortes et al., 2021). Unfortunately, in soccer training, coaches tend to focus on physical and tactical training and know little about the benefits of video-based perceptual-cognitive training. We outline the potential benefits of VBT for enhancing perceptual-cognitive skills, which will have practical implications for encouraging the use of video-based approaches in soccer training and improving training methods. From a practical standpoint, coaches can use VBT to complement regular soccer training, thus ensuring that players’ motor skills are fully developed.

### Strengths and limitations

We systematically reviewed the findings related to the effects of VBT on perceptual-cognitive skills in football, focusing on the development of anticipation and decision-making judgments in athletes with video-based perceptual-cognitive training. Overall, the available evidence tended to support the view that VBT is an effective method to improve athletes’ anticipation and decision-making skills. Although we outlined the potential benefits of VBT for perceptual-cognitive skills, it is important to consider the limitations of this review. First, we focused on the training of perceptual-cognitive skills for anticipation and decision-making. Therefore, results from training involving other perceptual-cognitive skills (e.g., visual search, pattern recognition) were excluded. Second, the lack of relevant data from the included studies prevented us from conducting a meta-analysis to quantify the effectiveness of the training. Finally, the language of the included studies was limited to English during the systematic database search, potentially missing some papers published in other languages.

### Perspectives and future direction

Based on the findings of this systematic review, we have several recommendations for future research. First, increasing the representativeness of tasks may be a useful direction. As technology develops, other methods, such as virtual video and 360VR, may be considered to be more representative of the actual on-field performance of athletes. Second, future research should also consider the activity conditions that occur in actual matches, such as fatigue, anxiety, and off-field noise. By introducing these potential factors that may affect match performance, the realism of the simulated task can be ensured. Finally, a key consideration when conducting VBT is transferring the effects of the training to on-court performance. Future research should actively design appropriate transfer tests for training tasks and conduct long-term transfer tests whenever possible to truly understand the benefits of perceptual-cognitive skills training.

## Conclusion

We systematically reviewed the findings of video-based perceptual-cognitive training to develop anticipation and decision-making skills in football players. Findings tended to support the view that VBT has advantages in developing anticipation and decision-making judgments in football players. Some findings are inconsistent with previous studies due to differences in intervention duration and experimental protocols, and follow-up studies are needed to improve the quality of the evidence. In addition, a formal meta-analysis of the existing studies to quantify the training effectiveness would be a valuable addition to future research.

## Author contributions

JZ and JM conceived the study. JZ and QG performed the literature search and screening, data extraction, and methodological quality assessment. JZ wrote the manuscript with the help of JM, QG and SZ. JM supervised the manuscript and accepted the grant. All authors involved in revising the manuscript and finalizing the final version, have read and agreed to the published version of the manuscript.
