# Supplementary material for: Effects of video-based training on anticipation and decision-making in football players: A systematic review
Source: Front Hum Neurosci. 2022 Nov 10;16:945067. doi: 10.3389/fnhum.2022.945067 (PMC9686440; doi:10.3389/fnhum.2022.945067)
Supplement: Supplementary file 1 [file Table_1.DOCX]

**Supplementary Table .** Quality assessment of the included studies.

| **Study** | **Item 1** | **Item 2** | **Item 3** | **Item 4** | **Item 5** | **Item 6** | **Item 7** | **Item 8** | **Item 9** | **Item 10** | **Item 11** | **Sum Scores** |
| --- | --- | --- | --- | --- | --- | --- | --- | --- | --- | --- | --- | --- |
| **Longitudinal studies** | | | | | | | | | | | | |
| Gabbett, et al., 2008 | 1 | 0 | 0 | 1 | 0 | 0 | 0 | 1 | 1 | 1 | 1 | 6 |
| Savelsbergh, et al., 2010 | 1 | 1 | 0 | 1 | 0 | 0 | 0 | 1 | 1 | 1 | 1 | 7 |
| Ryu, et al., 2013 | 1 | 1 | 0 | 1 | 0 | 0 | 0 | 1 | 1 | 1 | 1 | 7 |
| Murgia, et al., 2014 | 1 | 1 | 0 | 1 | 0 | 0 | 0 | 1 | 1 | 1 | 1 | 7 |
| Nimmerichter, et al., 2016 | 1 | 0 | 0 | 1 | 0 | 0 | 0 | 1 | 1 | 1 | 1 | 6 |
| Fortes, et al., 2021 | 1 | 1 | 0 | 1 | 0 | 0 | 1 | 1 | 1 | 1 | 1 | 8 |
| **Acute studies** | | | | | | | | | | | | |
| Poulter, et al., 2005 | 1 | 1 | 0 | 1 | 0 | 0 | 0 | 1 | 1 | 1 | 1 | 7 |
| OÑA, et al., 2009 | 1 | 0 | 0 | 0 | 0 | 0 | 0 | 1 | 1 | 1 | 1 | 5 |
| OÑA, et al., 2010 | 1 | 0 | 0 | 1 | 0 | 0 | 0 | 1 | 1 | 1 | 1 | 6 |
| ShafIzadeh, et al., 2012 | 1 | 1 | 0 | 1 | 0 | 0 | 0 | 1 | 1 | 1 | 1 | 7 |

Note: Item 1 = eligibility criteria; Item 2 = randomization; Item 3 = concealed allocation; Item 4 = similar baseline; Item 5 = blinding of all subjects; Item 6 = blinding of all therapists; Item 7 = blinding of all assessors; Item 8 = more than 85% retention; Item 9 = intention to treat analysis; Item 10 = between-group comparison; Item 11 = point measures and measures of variability; “0” = absent or unclear; “1” = clearly described. A higher score indicates better methodological quality.
